# Akt1 Mediates Neuronal Differentiation in Zebrafish via a Reciprocal Interaction with Notch Signaling

**DOI:** 10.1371/journal.pone.0054262

**Published:** 2013-01-14

**Authors:** Yi-Chuan Cheng, Fu-Yu Hsieh, Ming-Chang Chiang, Paul J. Scotting, Hung-Yu Shih, Sheng-Jia Lin, Hui-Lan Wu, Han-Ting Lee

**Affiliations:** 1 Graduate Institute of Biomedical Sciences, College of Medicine, Chang Gung University, Taoyuan, Taiwan; 2 Department of Life Science, Fu Jen Catholic University, New Taipei City, Taiwan; 3 Children’s Brain Tumour Research Centre, Centre for Genetics and Genomics, University of Nottingham, Queen’s Medical Centre, Nottingham, United Kingdom; Institute of Cellular and Organismic Biology, Taiwan

## Abstract

Akt1 is well known for its role in regulating cell proliferation, differentiation, and apoptosis and is implicated in tumors and several neurological disorders. However, the role of Akt1 in neural development has not been well defined. We have isolated zebrafish *akt1* and shown that this gene is primarily transcribed in the developing nervous system, and its spatiotemporal expression pattern suggests a role in neural differentiation. Injection of *akt1* morpholinos resulted in loss of neuronal precursors with a concomitant increase in post-mitotic neurons, indicating that knockdown of Akt1 is sufficient to cause premature differentiation of neurons. A similar phenotype was observed in embryos deficient for Notch signaling. Both the ligand (*deltaA*) and the downstream target of Notch (*her8a*) were downregulated in *akt1* morphants, indicating that Akt1 is required for Delta-Notch signaling. Furthermore, *akt1* expression was downregulated in Delta-Notch signaling-deficient embryos and could be induced by constitutive activation of Notch signaling. In addition, knockdown of Akt1 was able to nullify the inhibition of neuronal differentiation caused by constitutive activation of Notch signaling. Taken together, these results provide *in vivo* evidence that Akt1 interacts with Notch signaling reciprocally and provide an explanation of why Akt1 is essential for the inhibition of neuronal differentiation.

## Introduction

Akt, also known as protein kinase B (PKB), was first identified as the cellular homolog of the v-akt thymoma viral oncogene transduced by AKT8 and later found to be a serine/threonine kinase that is regulated through phosphatidylinositol-3-kinase (PI3K)-mediated signaling [1], [2], [3]. PI3K/Akt signaling is initiated when receptors such as receptor tyrosine kinases (RTKs) or G protein-coupled receptors (GPCRs) activate PI3K. PI3K then phosphorylates phosphatidylinositol 4,5-biphosphate (PIP2) to generate phosphatidylinositol 3,4,5-triphosphate (PIP3) which recruits Akt to the plasma membrane contributing to the conformational change of Akt. Once positioned at the membrane, Akt become activated upon phosphorylation by phosphoinositide-dependent kinase-1 (PDK1) and mammalian target of rapamycin complex 2 (mTORC2). Activated Akt phosphorylates a variety of substrates, which contribute to diverse cellular roles, including cell survival, growth, proliferation, migration, and angiogenesis, and various aspects of metabolism [4]. Aberrant Akt signaling was found in several human diseases, ranging from cancer to metabolic dysfunction and mental diseases [5]. To date, three mammalian isoforms, Akt1 (PKBα), Akt2 (PKBβ), and Akt3 (PKBγ), have been identified; all three share a high degree of sequence and structural similarities [6]. These Akt isoforms show differences in tissue-specific expression patterns and play distinct physiological roles with some overlapping functions [5], [7], [8].

Akt1 is the founding member and the most intensively studied protein of the family. Mutations in Akt1 that result in constitutive activation or elevated Akt1 expression have been implicated in a wide range of cancers, including colorectal, breast, prostate, lung, pancreatic, liver, and ovarian cancers as well as in leukemia and glioblastomas [9], [10], [11]. Mutations in this gene have been associated with Proteus syndrome [12] and schizophrenia [13]. In the nervous system, extensive studies have shown that Akt1 plays crucial roles in multiple cellular processes, including neural cell survival [14], [15], [16], [17], [18] and enhancement of the proliferation of neural progenitors [19], and is required for axon growth [20], [21]. Controversial results showed Akt1 could both promote [22], [23] and inhibit neuronal differentiation [24], [25]. However, most of these data were obtained from *in vitro* analyses, and experimental approaches mainly rely on the mis-expression of constitutive active or dominant negative constructs, which may not reflect the physiological role of Akt1. In addition, abundant expression of *Akt1* was detected in the developing nervous system [26]. Nonetheless, very few studies have described the prenatal role of Akt1 *in vivo*, and the defect in the developing nervous system of *Akt1* knockout mice has not been thoroughly described [26], [27], [28]. However, altered expression of genes involved in neuronal development was detected in the prefrontal cortex of *Akt1*-deficient mice analyzed by transcriptional profiling [29]. Further *in vivo* evidence was obtained by over-expression of the constitutively active form of human Akt1 in *Xenopus* embryos, which induced neuronal markers (shown by RT-PCR) [30]. These studies provide a strong indication that Akt1 participates in neural development, but its endogenous role in neural development remains unclear.

The zebrafish, *Danio rerio*, has emerged as an excellent vertebrate model, with advantages over other organisms for the study of many aspects of developmental biology. In particular, the morpholino approach provides efficient and economic phenocopies of gene mutations. Here, we isolated the previously uncharacterized zebrafish *akt1* gene and show that it is expressed in the developing nervous system. Knockdown of endogenous Akt1 resulted in apoptosis and premature differentiation of neuronal precursors. We further showed that Akt1 reciprocally interacts with the Delta-Notch-Su(H) signaling cascade, which is essential for the inhibition of neuronal differentiation. Our *in vivo* data provide further insights into the roles of Akt1 in neural development and in Notch-regulated neuronal differentiation.

## Results

### Identification and Isolation of Zebrafish *akt1*

BLAST analysis using human AKT1 amino acid sequence as a template failed to identify any putative homologue from current zebrafish databases (the zebrafish genome sequence is only 87% complete). We therefore performed PCR from a home-made zebrafish cDNA library (a kind gift from Sheng-Ping L. Hwang) using degenerate primers designed from the mammalian AKT1 protein sequence, and isolated a cDNA clone containing a fragment that showed high similarity to mammalian *AKT1*/*Akt1*. Sequence comparison to mammalian homologues revealed that this fragment was not a full coding region. The missing 5′-end sequence was then amplified by 5′ rapid amplification of cDNA ends (5′ RACE) and sequenced. Primers were then designed accordingly to amplify the full open reading frame from total cDNA extracted from zebrafish embryos. The product of the PCR comprised 1425 bp, encoding a 475-residue peptide containing putative start and stop codons matching approximately the same positions as in other vertebrate *AKT1*/*Akt1* genes (Fig. S1A). This fragment shows structural features of the Akt family members including a pleckstrin homology (PH) domain, a kinase domain and a hydrophobic motif. The putative coding sequences showed the highest degree of similarity to the AKT1 homologues, with 90% identity and 95% similarity to chicken AKT1 and 89% identical and 94% conserved amino acids compared with human and mouse AKT1. Phylogenetic analysis also showed this fragment was most closely related to mammalian AKT1 (Fig. S1B). A conserved threonine at position 302 and a serine at position 467 (308 and 473 in mammals, respectively), which have been shown to be phosphorylated by PDK1 and mTORC2, respectively, in other homologues [31], were also identified (Fig. S1A). Besides these two sites, residues tyrosine 309, tyrosine 320 and threonine 444 (315, 326 and 450 in human, respectively), which have been shown to be involved in the regulation of Akt activity, are also conserved in this zebrafish fragment [32] (Fig. S1A). Alignment with the genomic sequence showed the open reading frame was contained within 13 exons and comparison of the exon-intron structure of the vertebrate *Akt1* homologues revealed that all intron positions are strictly conserved (arrowheads in Fig. S1A). This provides strong evidence for their evolution from a common ancestor gene specific to this subgroup. This result further substantiates the orthology of this gene homologous to mammalian *AKT1*/*Akt1*. We therefore named this gene zebrafish *akt1* (GenBank accession number JX307852). Teleost fish often have two co-orthologues due to an additional genome-wide duplication event, which contrasts with the single gene copy found in humans and other mammals [33]. The zebrafish genome has not been fully sequenced, so we screened the genomic databases of other teleost fishes with complete genome sequences (medaka, fugu, and *Tetraodon*). We identified only one *akt1* homologue in each species (data not shown), which suggests that *akt1* is not duplicated in teleost fish and that the zebrafish genome contains only one *akt1* gene.

### Zebrafish *akt1* is Expressed in the Developing Nervous System

**Figure 1:**
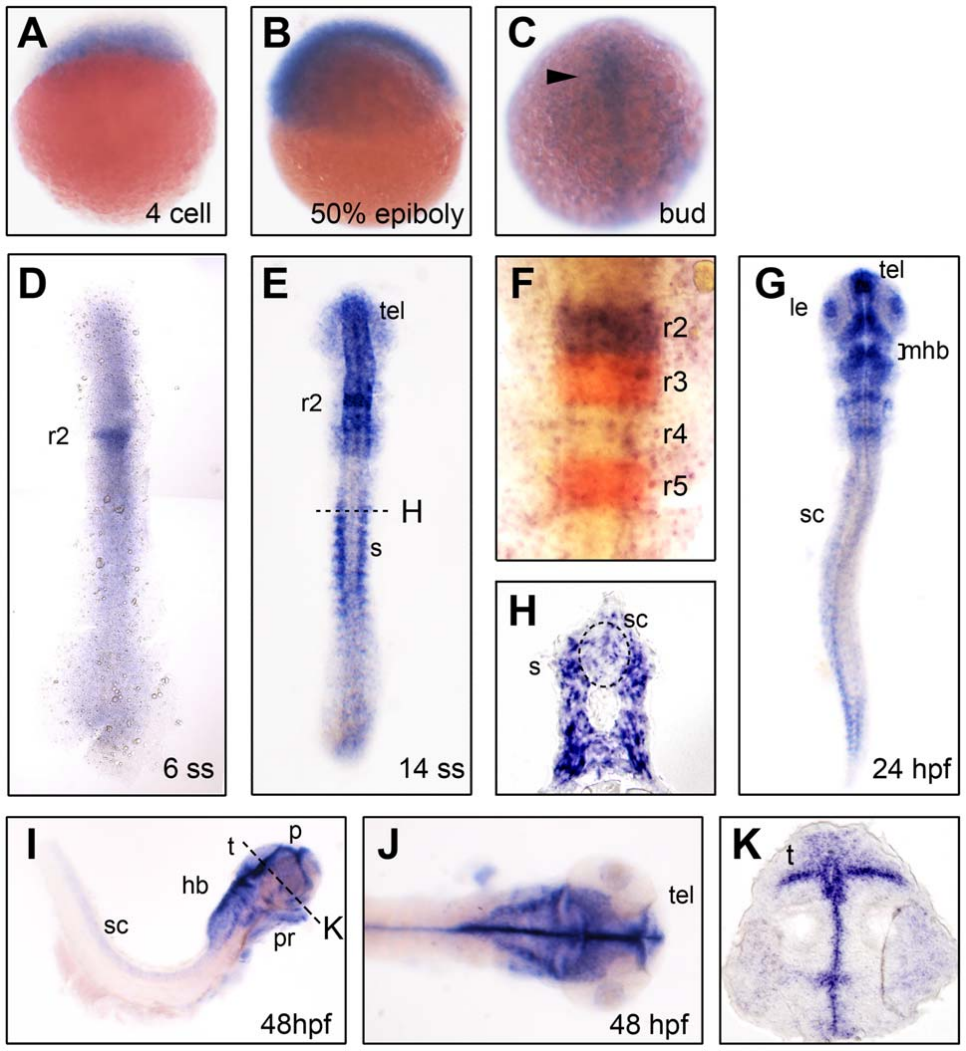
*akt1* expression in the developing zebrafish. *akt1* expression was detected by *in situ* hybridization in the developing nervous system during zebrafish embryogenesis. The embryo stages are shown in the bottom right corner of each panel. (C–F and G) Dorsal view with anterior to the top. (I) Lateral view with anterior to the right. (J) Dorsal view with anterior to the right. *akt1* expression appears first in the developing nervous system during the bud stage (arrowhead in C) and later becomes restricted to specific brain areas (D–K). Relatively weak expression was detected in the spinal cord from the bud stage (C) and persisted until the final stage that was analyzed (48 hpf) (C–E and G–J). (F) *akt1* expression (purple) in rhombomere 2 analyzed by double *in situ* hybridization with *krox20* (red). H and K are cross-sections from E and I, respectively. hb, hindbrain; le, lens; mhb, midbrain-hindbrain boundary; p, pallium; pr, pharyngeal arches; r2–r5, rhombomere 2–5; s, somites; sc, spinal cord; t, tectum; tel, telencephalon.

Expression of *akt1* was analyzed by whole-mount *in situ* hybridization. Ubiquitous expression through the whole embryo before gastrulation suggests that the gene is expressed as a maternal mRNA (Fig. 1A–B). Widespread low-level expression in the developing central nervous system could be detected from the tail bud stage, with strong expression observed in the brain from the 6-somite stage until the latest stage analyzed (48 hours post fertilization [hpf]; Fig. 1C–H), whereas expression in the spinal cord was relatively weak (Fig. 1C–H). Much higher levels of *akt1* expression could be observed in the hindbrain from the 6-somite stage (Fig. 1D) and in the telencephalon from the14-somite stage (Fig. 1E). In addition to the developing nervous system, significant expression could be seen in the somitic furrows from the 14-somite stage to 24 hpf, and transient expression in the developing lens at 24 hpf and in the pharyngeal arches at 48 hpf (Fig. 1D–H). In comparison, mouse *Akt1* is also strongly expressed in embryonic brain and muscular derivatives [26], which shows partial conservation with the expression of zebrafish *akt1*; however, the mouse homologue is also transcribed in heart, lung, thymus and skin, where no expression of zebrafish *akt1* was detected during the stages analyzed.

### Akt1 Knockdown Causes Developmental Abnormalities

**Figure 2:**
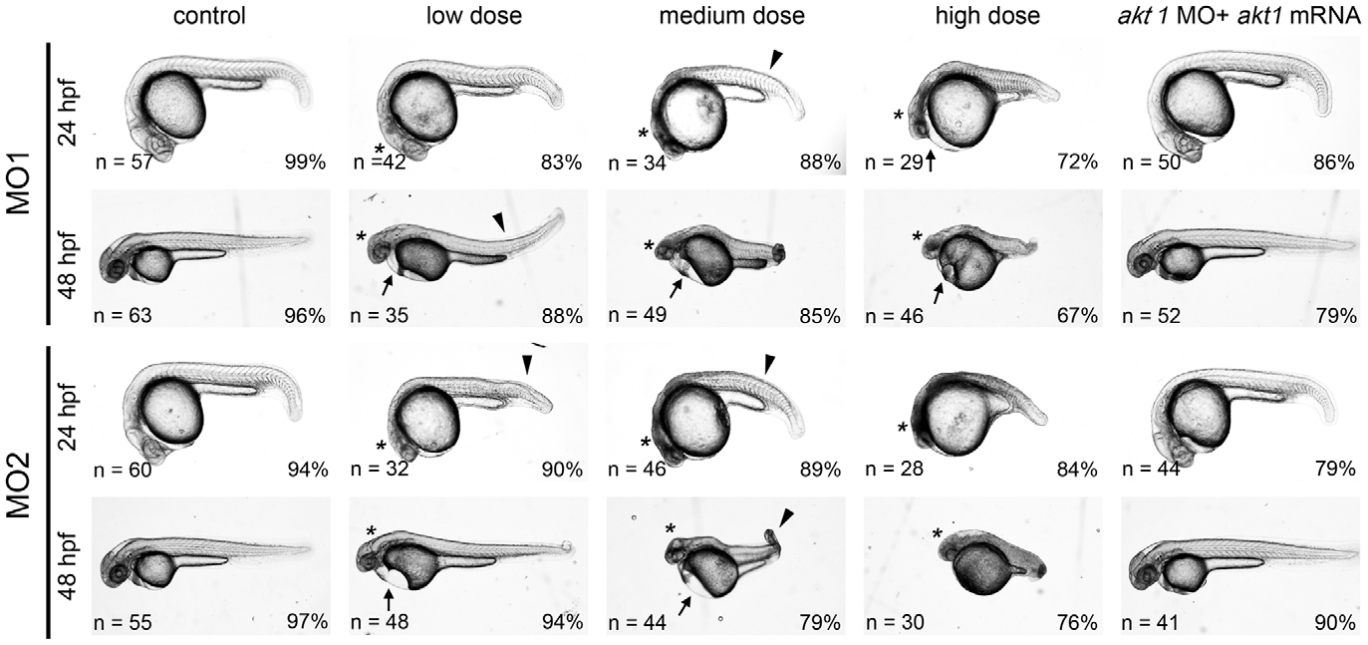
Akt1 knockdown using specific morpholinos is sufficient to cause developmental abnormalities in a dose-dependent manner. Representative images of morpholino-injected embryos. The following doses were used for microinjection: low dose, 6 ng MO1 or 1 ng MO2; medium dose, 10 ng MO1 or 2 ng MO2; high dose, 14 ng MO1 or 4 ng MO2. Arrowheads denote bent body characteristics, while arrows indicate edema in the pericardial sac. Brain malformations are marked by asterisks. These morphological phenotypes were rescued by concomitant injection with 1 ng *akt1* mRNA, as shown in the right panels.

In order to delineate the role of Akt1 in neurodevelopment, the morpholino (MO) knockdown approach was used to interfere with its expression. An antisense morpholino was synthesized to target the translation start site of *akt1* mRNA to block protein production (MO1), and a second morpholino (MO2) was designed that targets the intron 9 - exon 10 boundary resulting in defective splicing and a truncated product (Fig. S2A). Comparison with sequences of other *akt* paralogues predicted binding of both morpholinos specifically to *akt1*. The specificity of *akt1* MO1 and MO2 was confirmed by rescue experiments in which the MOs were coinjected with mRNA for *akt1*, as described for each experiment below. To confirm the efficacy of the morpholino knockdown approach, *akt1* MO1 was coinjected with the mRNA of a ‘reporter construct that contained the *akt1* MO1 binding sequence upstream of an enhanced green fluorescent protein reporter (5′*akt1-EGFP*). Effective knockdown of the translation of this construct (evident by loss of EGFP protein) was observed upon coinjection with *akt1* MO1 but was unaffected by coinjection with control morpholino (Fig. S2D). To confirm the efficacy of *akt1* MO2, RT-PCR with primers that spanned the MO2 binding sequence was employed and a fragment corresponding to an mRNA lacking the 215 bp of exon10 was detected in the morpholino injected embryo extract (Fig. S2B). Sequencing of the mis-spliced product confirmed exon 10 deletion and further showed a predicted premature stop codon in the kinase domain that would truncate the encoded protein and resulted in the loss of the phosphorylation sites T444 and S467 that have been shown to be required for Akt1 activation (Fig. S1A, C). Injection of MO1 or MO2 caused identical morphological defects, including brain malformation, a bent trunk and pericardial edema, in a dose-dependent manner, analyzed at 24 hpf and 2 days post fertilization (dpf) (Fig. 2). Concomitant injection of morpholinos with *akt1* mRNA rescued these phenotypes, indicating that the morpholino-induced defect was due to loss of Akt1 function (Fig. 2). We used 6 ng MO1 or 1 ng MO2 for the following experiments since these doses were sufficient enough to caused specific knockdown of Akt1 without severe morphological defects.

### Knockdown of Akt1 Increases cell Apoptosis via a p53-independent Mechanism

**Figure 3:**
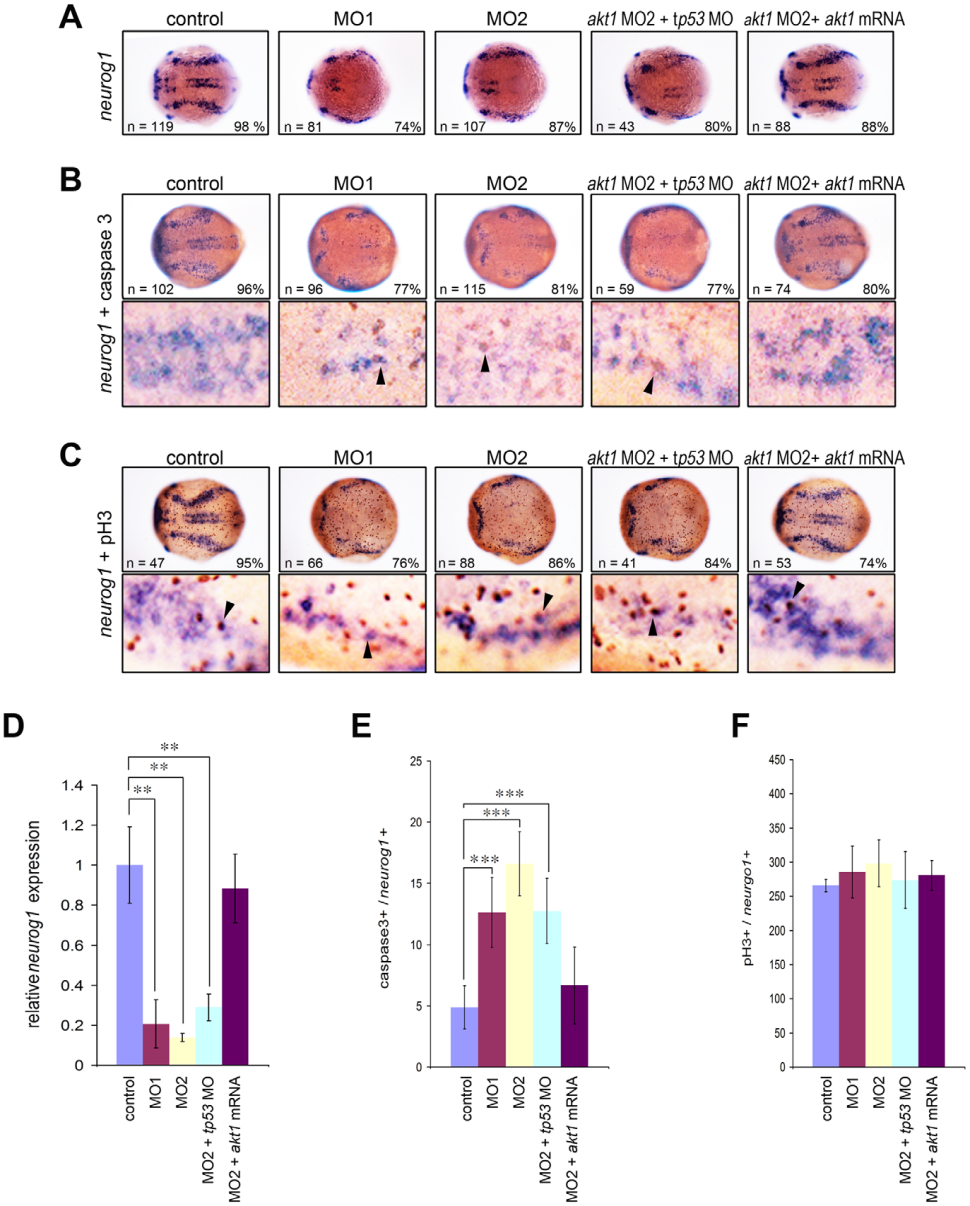
Decreased neuronal precursors in Akt1 knockdown embryos during early neurogenesis. (A) *In situ* hybridization showing the dramatic downregulation of *neurogenin1* in *akt1* morpholino-injected embryos. This effect was rescued by co-injection with *akt1* mRNA but not a *tp53* morpholino, which was confirmed by qPCR analysis (D). (B) Apoptotic cells were detected using caspase-3 antibody (brown) and double-labeled with *neurogenin1* (purple), which showed an increase in apoptotic cells in *akt1* morphants. The double-labeled cells are indicated with arrows and quantified in E. (C) Proliferating neurons were double-labeled with phosphohistone H3 antibody and *neurogenin1* riboprobes (arrows), which showed no significant difference between *akt1* morphants and controls. This was confirmed by quantification (F). In A–C the lower panels are enlargements of the upper panels. **, *p*<0.01; ***, *p*<0.001.

We first analyzed the effect of Akt1 knockdown in the developing nervous system by examining *neurogenin1*-positive neuronal precursors during early neurulation. Whole-mount *in situ* hybridization showed that expression of *neurogenin1* was dramatically decreased in the morpholino injected embryos at bud stage, which could be rescued by *akt1* mRNA injection (Fig. 3A). Quantitative real time PCR (qPCR) analysis confirmed a 5-fold decrease in *neurogenin1* expression in *akt1* morphants (Fig. 3D). These results indicate that Akt1 was required for the formation of neuronal precursors. Since previous *in vitro* studies showed Akt1 is able to mediate neural cell survival and to suppress apoptosis [18], [34], we suspected morpholino knockdown of Akt1 reduced the number of neuronal precursors through induced apoptosis or inhibition of proliferation. To investigate whether the loss of *neurogenin1*-positive neurons was due to cell death, apoptotic neurons were analyzed by looking for the presence of proteolytic activation of the effector caspase-3 by immunohistochemistry on *akt1* morphants. Following *in situ* hybridization with *neurogenin1* RNA probe, embryos were subjected to immunohistochemical staining to determine whether apoptosis was localized to neuronal precursors (Fig. 3B). Injection of *akt1* morpholino resulted in slightly increased activated caspase-3-positive signals in comparison with the controls (Fig. 3B, E), suggesting that Akt1 was required for the survival of neurons. However, previous studies showed that some morpholinos can cause off-target apoptosis mediated by p53 activation [35]. To rule out this possibility, we co-injected a *tp53* morpholino with *akt1* morpholino which did not affect the *akt1* morphant phenotype (Fig. 3A, B). Taken together, these results showed that knockdown of Akt1 results in neuronal apoptosis and suggest that Akt1 contributes to neuronal survival through a mechanism alternative to p53.

Previous *in vitro* studies showed AKT1 could regulate the proliferation of neural progenitors [19]. We therefore analyzed proliferating neurons using phosphohistone H3 antibody and counterstained with *neurogenin1*. The results demonstrated that neural proliferation remained intact after Akt1 knockdown (Fig. 3C, F), indicating that Akt1 was not required for the proliferation of neurons.

### Loss of Akt1 is Sufficient to Cause Precocious Neuronal Differentiation

**Figure 4:**
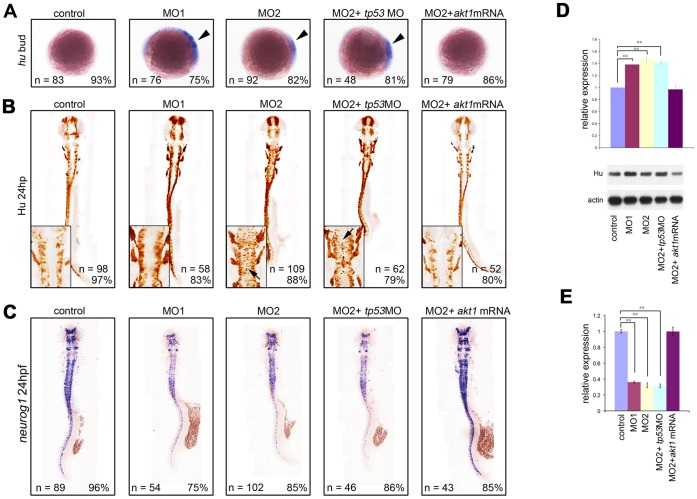
Akt1 morphants exhibit upregulation of HuC/D expression with simultaneous downregulation of *neurogenin1* expression. (A) During the bud stage, injection with *akt1* morpholinos upregulated the expression of *elavl3* compared with the controls. (B) HuC/D-expressing post-mitotic neurons increased massively in *akt1* morpholino-injected embryos, as shown by immunohistochemical analysis with anti-Hu antibody at 24 hpf. Note the ectopic HuC/D expression in the ventricular zone (arrows in insert panels). (C) At the same stage, *neurogenin1* expression was reduced significantly by Akt1 knockdown. (D) Levels of HuC/D expression were confirmed by Western blot analysis and are quantified in the top panel. (E) qPCR analysis further confirmed the decreased expression of *neurogenein1* at 24 hpf (C) in *akt1* morpholino-injected embryos. All phenotypes could be rescued by concomitant injection with *akt1* mRNA but not a *tp53* morpholino. **, *p*<0.01.

Given the reduced number of neuronal precursors following knockdown of Akt1, we investigated whether this might be because it elicited premature differentiation of neurons. The expression of *elavl3* (encoding HuC), which marks the neurons of late determination and differentiation [36], was therefore investigated in Akt1 knockdown embryos. *elavl3* transcripts were not detectable at bud stage in wild-type embryos, and emerged only from the 3 somite-stage, reflecting the stage of the beginning of neuronal differentiation (Fig. 4A, B). On the contrary, a significant amount of *elavl3* transcripts was observed in Akt1 knockdown embryos at bud stage, concurrent with reduced *neurogenin1*-positive neuronal precursors (Fig. 4A–B; compare with Fig. 3A), indicating Akt1 knockdown caused premature differentiation of neurons. We further investigated the effect of Akt1 knockdown using a post-mitotic neuronal marker, HuC/D antibody, on embryos at 24 hpf. The results showed that embryos injected with *akt1* morpholino exhibited significant upregulation of HuC/D expression (Fig. 4A, C). Notably in the normal developing hindbrain, the differentiating neurons were normally confined to the center and bilateral sides of each rhombomere; in contrast, ectopic mature neurons filling nearly the entire hindbrain were observed in the *akt1* morphants (insert panels in Fig. 4A). Ectopic HuC/D-positive cells were also observed in several regions where normal neuronal differentiation would not occur (arrows in Fig. 4B). This effect was confirmed by Western blot analysis showing a 1.4-fold increase in HuC/D expression in *akt1* morphants (Fig. 4D). Concurrently with the increased HuC/D expression, the expression of *neurogenin1* was significantly downregulated at 24 hpf (Fig. 4A), indicating that neurons were prematurely differentiated from *neurogenein1*-positive precursors into HuC/D-positive differentiating neurons. The effect of *akt1* morpholino could be attenuated by co-injection with *akt1* mRNA but not *tp53* morpholino (Fig. 3 and Fig. 4). Taken together, these results indicate that Akt1 is required for the inhibition of neuronal differentiation and that this function is separable from neuronal survival.

### Akt1 Interacts with Notch Signaling to Mediate Neuronal Differentiation

**Figure 5:**
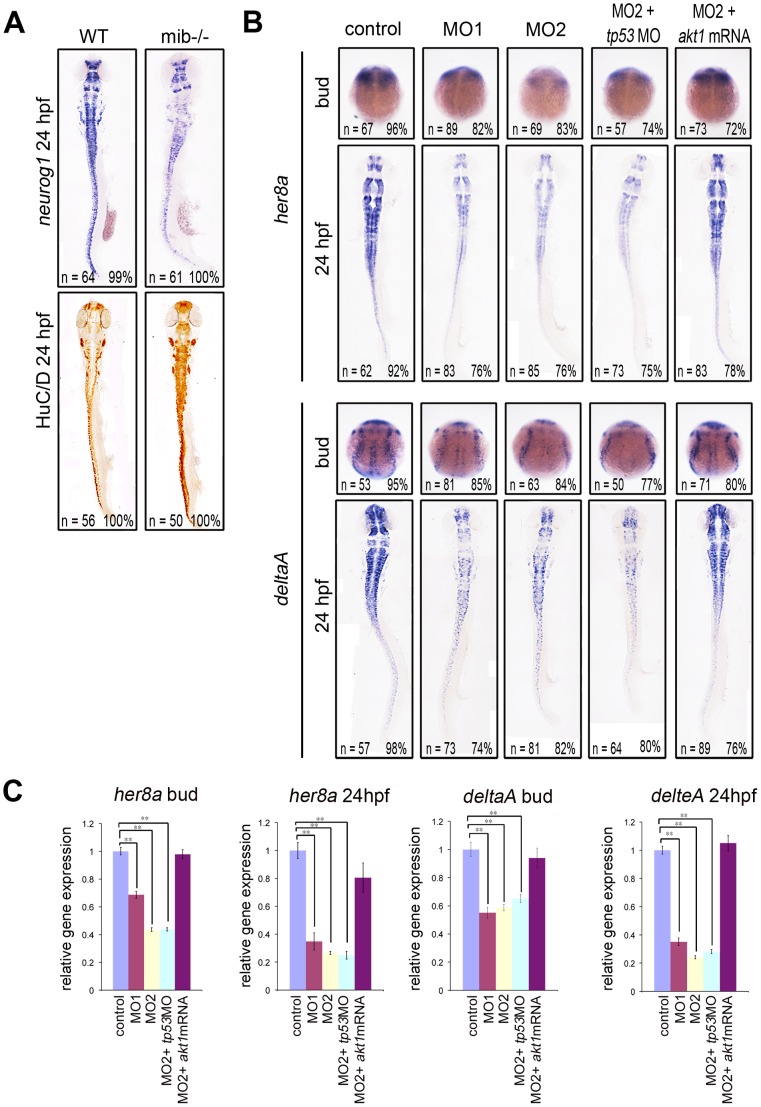
Akt1 is required for the expression of *her8a* and *deltaA*. (A) *neurogenin1* expression was downregulated whereas HuC/D expression was upregulated in *mib^ta52b^* mutants (right) compared with wild-type siblings (left). (B) *In situ* hybridization showed that *her8a* and *deltaA* were expressed at lower levels in *akt1* morphants compared with the controls during the bud stage and at 24 hpf. This phenotype was restored by coinjection with *akt1* mRNA but not with a *tp53* morpholino. (C) The expression level in B was confirmed by qPCR. **, *p*<0.01.

Recent studies have shown that the Akt and Notch signaling pathways interact through complex molecular interactions in the normal developing nervous system and in neoplastic tissues [37]. Notch signaling is initiated upon interaction with its ligands Delta or Serrate/JAGGED and consequently trigger the transcription of target genes, *Hairy* and *Enhancer-of-split* [*H/E(Spl)*], which suppress the expression of proneuronal genes and inhibit neurogenesis [38]. Defects in Notch signaling result in premature differentiation of neurons [39]. This phenotype of *akt1* morphants resembled the effect observed in Notch-deficient embryos [40] (Fig. 4A and Fig. 5A), suggesting a potential functional interaction between Akt and Notch signaling pathways. To investigate whether these two signals interact to regulate neuronal differentiation, we first analyzed in *akt1* morphants the expression of *her8a*, a direct downstream target of Su(H)-dependent Notch signaling in zebrafish, which is essential for the inhibition of neuronal differentiation [41]. This showed that *her8a* expression was downregulated in Akt1 deficient embryos (Fig. 5B, C), suggesting that Akt1 was essential for *her8a* transcription. We next sought to examine whether Akt1 was required for the initiation of Notch signaling by analyzing the ligand of Notch signaling, DeltaA. Injection of *akt1* morpholino also downregulated the expression of *deltaA* (Fig. 5B–C), suggesting that Akt1 is an upstream mediator that is essential for Notch signaling.

**Figure 6:**
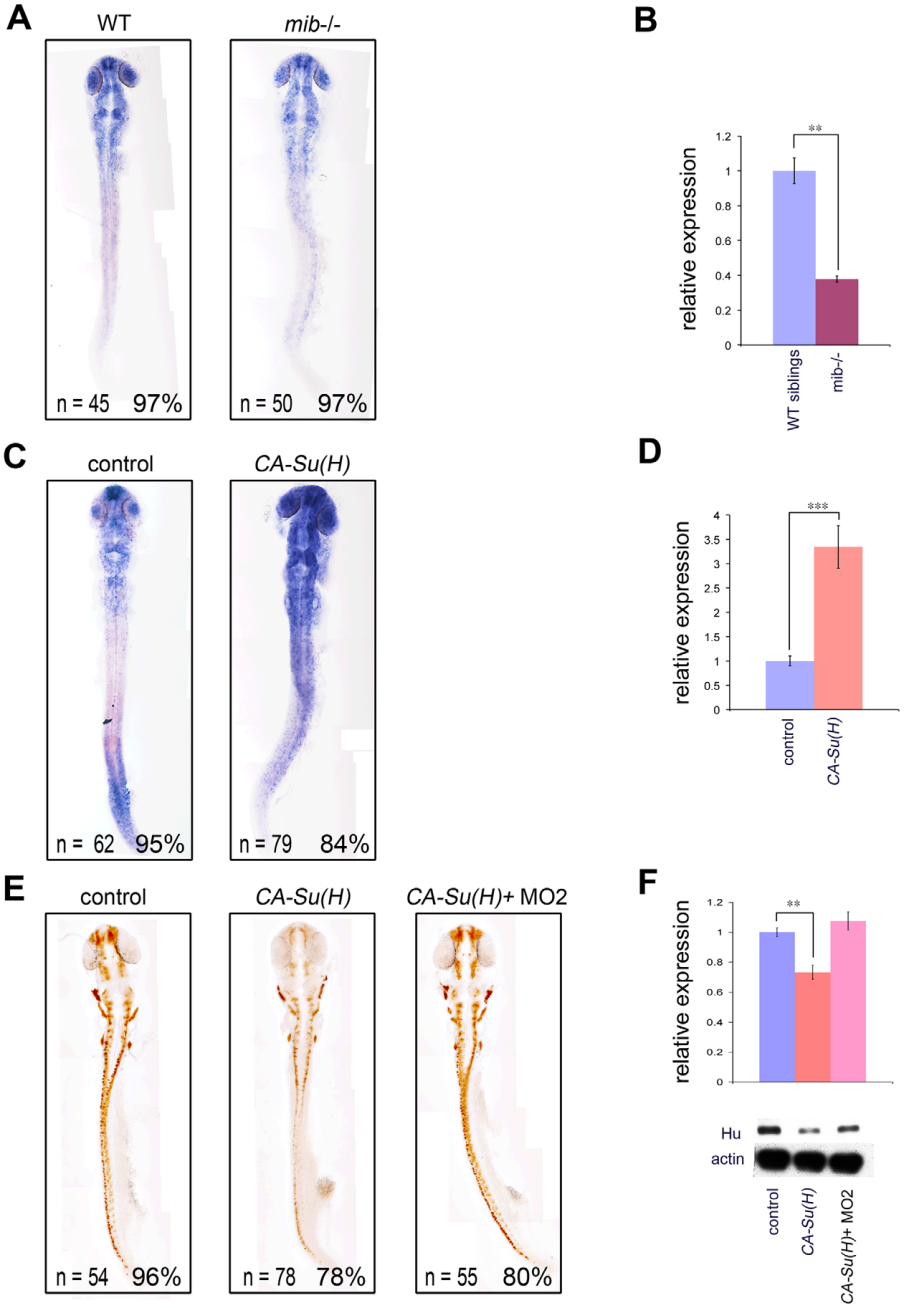
Notch-Su(H) signaling is sufficient to induce *akt1* expression and inhibit neuronal differentiation. (A) *akt1* expression was downregulated in Notch activity-deficient *mind bomb* mutant embryos compared with wild-type siblings as shown by *in situ* hybridization, which was confirmed by qPCR analysis in B. (C) *akt1* expression levels were upregulated by constitutive-active *Su(H)* injection and quantified by qPCR in D. (E) Injection of constitutive-active *Su(H)* significantly downregulated HuC/D expression and this phenotype was nullified by *akt1* knockdown. (F) Western blot analysis confirmed the levels of HuC/D expression shown in E. CA-Su(H), constitutive-active Su(H); **, *p*<0.01; ***, *p*<0.001.

Our results are consistent with previous *in vitro* studies showing that Akt signaling can act as a positive upstream regulator for Notch signaling [42], [43], [44]. Conversely, several reports also showed that Akt can be regulated by Notch signaling [37], [45], [46], [47]. To further substantiate the role of Akt1 in Notch signaling, we analyzed the expression of *akt1* in *mind bomb* mutant embryos (*mib^ta52b^*) that have a strong Notch pathway deficiency [48] due to mutation of a ubiquitin ligase required for Delta ligand activity [49]. The expression of *akt1* was downregulated in *mib^ta52b^* mutants (Fig. 6A), indicating that Notch activation is required for *akt1* transcription in turn.

The Notch receptor interacts with its canonical ligands Delta and Serrate, which trigger proteolytic cleavage that releases the Notch intracellular domain (NICD) to enter the nucleus. NICD then interacts with the DNA-binding protein CSL [CBF/RBP-J, Su(H), LAG-1], leading to the transcription of target genes [38]. To further test whether *akt1* is activated through the Su(H)-dependent Notch pathway, we expressed mRNA encoding a constitutively active form of *Su(H)* that has been shown to activate Notch signaling and its target genes [50]. This resulted in upregulation of *akt1* expression (Fig. 6B), indicating that *akt1* can be induced by Su(H)-dependent Notch signaling. Taken together, our results demonstrated that the expression of *akt1* can be upregulated by the DeltaA-Notch-Su(H) cascade.

Activation of Notch signaling by over-expressing constitutive-active *Su(H)* caused downregulation of HuC/D due to constitutive inhibition of neuronal differentiation [40], [50] (Fig. 6C). This phenotype is the converse of what we observed in Notch signaling and Akt1-deficient embryos. To further confirm that Akt1 expression is essential for Notch signaling-mediated inhibition of neuronal differentiation, we constitutively activated the Notch pathway by injection of the dominant-active form of *Su(H)* at the one-blastomere stage followed by injection of *akt1* morpholino at -blastomere stage, and analyzed the effect of neuronal differentiation. The results showed that the effects caused by dominant-active *Su(H)* was nullified by Akt1 knockdown (Fig. 6C–D), indicating that Akt1 is required for Su(H)-dependent Notch signaling-mediated inhibition of neuronal differentiation. Taken together, the results suggest that Akt1 mediates Notch signaling in a positive feedback loop and is essential for the inhibition of neuronal differentiation.

## Discussion

Akt1 is well known for its potential role in oncogenesis and is associated with several neurological defects, such as familial schizophrenia [13] and dopamine-associated neuropsychiatric disorders [51]. Despite its pathological importance and enriched expression in the developing nervous system, however, the role of Akt1 in neurodevelopment has remained unclear. Most previous studies of Akt1 have relied on the expression of mutant constructs in neural cells that might result in non-physiologically relevant effects. In the present study, we found that the expression of zebrafish *akt1* is generally similar to mammalian *AKT1*/*Akt1*; in particular, it is abundantly expressed in the developing nervous system. The spatial distribution of *akt1* transcripts revealed its potential role in neuronal differentiation. Morpholino-induced knockdown of endogenous Akt1 caused precocious generation of early-differentiating neurons, leading to depletion of precursors. To our knowledge, this is the first study to reveal that downregulation of Akt1 is sufficient to cause premature neuronal differentiation. Consistent with this observation, overexpression of Akt1 inhibited neuronal differentiation, while transfection of the dominant inhibitory mutant form of Akt1 accelerated NGF-induced neuronal differentiation in PC12 cells [24]. Taken together, these results indicate that Akt1 is required for the inhibition of neuronal differentiation. On the contrary, other *in vitro* studies demonstrated that Akt1 is a positive regulator of neuronal differentiation [22], [52], and recent *in vivo* studies also showed that Akt1 induced the expression of neural markers in *Xenopus* [30] and promoted the differentiation of GABAergic neurons in mouse [23]. This seemingly contradicts our results. However, since these studies were performed by over-expressing a constitutively active form of human AKT1, we suggest that this may trigger abnormal activation of a mechanism yet to be identified in neuronal differentiation. An alternate explanation is that the function of Akt1 may be strongly influenced by the cell type and developmental stage in which it is expressed.

We demonstrated that Akt1 regulates neuronal differentiation through crosstalk with Notch signaling. Although interaction between Akt and Notch signaling has been reported, how Notch signaling responds to Akt activation remains highly controversial. Some studies have shown that components of Akt signaling, such as PI3K, mTOR, and Akt itself, positively regulate Notch signaling in cells ranging from murine fibroblasts and neurons to a variety of human tumor cells [43], [44], [53], [54]. Conversely, some studies report that Akt signaling negatively regulates Notch signaling [55], [56]. These observations indicate that the response of Notch signaling to Akt activation is highly regulated both temporally and spatially. Here, we focused on differentiating neuronal precursors and showed that Akt1 is required for the activation of Notch signaling, and that this positive regulation results in inhibition of neuronal differentiation. Previous *in vitro* studies showed Akt1 activation is required for the transcription of *Delta-like 4* ligand [42], *Notch 1* receptor [53], and *Hes1* transcription factor [43]. Consistent with these data, we also found that knockdown of Akt1 downregulated the transcription of *deltaA* and *her8a*. However, it remains to be confirmed whether Akt1 mediates Notch signaling in a direct or indirect manner and how Akt1 regulates the components of Notch signaling in neural development.

Akt signaling can also be regulated by Notch signaling, as has been described in several different cellular models. However, the underlying molecular mechanism is not clear. Previous studies in several cell lines revealed that Notch signaling positively regulates Akt signaling [45], [46], [57] and further analysis showed that Akt could be activated by the direct target of Notch, HES1 [47]. In concordance with these previously data, we showed that *akt1* expression was downregulated in Notch-deficient embryos. Furthermore, we constitutively activated Notch signaling by injection of an active form of *Su(H)* and found this was sufficient to increase the transcription of *akt1*. This is an intriguing result since most previous studies showed that Notch mediates Akt signaling by inhibiting PTEN, a phosphatase that dephosphorylates PIP3, and results in inhibition of the AKT signaling pathway and consequently activates Akt. Our results therefore reveal a novel mechanism in which Notch signaling is sufficient to upregulate Akt1 at the transcriptional level rather than by phosphorylation of the Akt1 protein.In conclusion, our results lead us to postulate the operation of a reciprocal regulatory loop between Akt and Notch signaling and provide an explanation of how Akt1 is required for the inhibition of the differentiation of neuronal precursors in zebrafish.

## Materials and Methods

### Ethics Statement

All experiments were performed in strict accordance to standard guidelines for zebrafish work and approved by the Institutional Animal Care and Use Committee of Chang Gung University (IACUC approval number: CGU05-05 and CGU08-86).

### Fish Maintenance and Mutants

*Tü* (wild type) and *mib^ta52b^* mutant zebrafish embryos were purchased from the Zebrafish International Resource Center (ZIRC, Oregon, USA) and were raised, maintained and paired under standard conditions. The embryos were staged according to the number of somites, hours post fertilization and days post fertilization [40].

### Sequence Comparisons and Phylogeny

Amino acid sequences were aligned and displayed using the Vector NTI (Invitrogen). Phylogenetic tree calculation was performed with ClustalX [39]. The GenBank accession numbers of the compared proteins are as follows: human AKT1 (NM_005163.2), AKT2 (NM_001626), AKT3 (NM_005465); mouse AKT1 (NM_009652), AKT2 (NM_001110208), AKT3 (NM_011785); chicken AKT1 (NM_205055), AKT3 (ENSGALP00000031235); zebrafish Akt2 (NM_198146.2), Akt2l (NM_212815.1), Akt3 (NM_001197201.1).

### cDNA Library Screening and Constructs Generation

Degenerate primers 5′- TA(CT)(CT)T(ACGT)CA(CT)TC(ACGT)GA(AG)AA(AG)AA(CT) -3′ and 5′- GC(ACGT)GT(ACGT)CC(ACGT)G(AT)(ACGT)GC(ACGT)G(AT)(AG)TA -3′) were designed according to the mammalian sequences to specifically amplify *akt1* fragment. A zebrafish 72 hour post fertilization cDNA library (8.7 pfu/ml in the λZAPII vector) was screened for the presence of *akt1* containing cDNA clone by PCR and the products were subjected for sequencing as described previously [58]. The rapid amplification of the 5′ cDNA end (5′ RACE) was performed using the 5′ RACE kit (Ambion) according to the manufacturer’s instructions. The open reading frame of *akt1* was PCR amplified with the primers 5′-GAATTCGCCACCATGGCGACAGATGTGGTGATCG-3′and 5′-GGAATTCCTCATGCTGTTCCGCTGGCCG-3′, which introduce *EcoR*I restriction sites suitable for cloning. The PCR product was digested with *EcoR*I and cloned into the pCS2^+^ vector. PCR amplifications were performed with the high fidelity Pfu polymerase (Promega) and constructs were sequenced to check for the absence of mutations.

### RNA and Morpholino Injection

Capped RNA encoding the full coding sequence of Akt1 and constitutive-active Su(H) was prepared as described previously [59]. The Su(H) constructs were kindly provided by Chris Kintner. Antisense morpholino oligonucleotides were purchased from Gene Tools, LLC (Oregon, USA). Two morpholinos against *akt1* were used: MO1 (GGAACGAGTCTTCACACGGGTCACC) that overlaps the ATG start codon, and MO2 (AGCACCTGCACACACACACATGTAC) that corresponds to intron9-exon10 boundary region sequence. A *tp53* morpholino with the sequence GACCTCCTCTCCACTAAACTACGAT (Gene Tools, LLC) was used. A control morpholino designed to a random sequence of nucleotides not found in the zebrafish genome (5′- CCTCTTACCTCAGTTACAATTTATA-3′; Gene Tools) or a morpholino with 5 bases mismatch to MO2 (5′- AGgACCTcCACAgACAgACATcTAC-3′; mismatched bases are indicated by small letters) was injected in an equal amount of MO2 as a control experiment (Figure S3). All injections were performed at the one to two-cell stage and mRNAs or morpholinos were introduced into blastomeres except where otherwise noted.

### Histological Analysis

Digoxigenin-UTP or Fluorescein-UTP labeled riboprobes to detect *akt1*, *deltaA*, *her8a*, *neurogenin1*, *krox20* and *elavl3* transcripts were synthesized according to manufacturer’s instructions (Roche), and *in situ* hybridizations were performed as described previously [40]. The color reaction was carried out using NBT/BCIP substrate (Roche) or Fast Red TR/Naphthol AS-MX (Sigma). For immunohistochemistry, the embryos were blocked in 5% goat serum and incubated with mouse anti-HuC/HuD monoclonal 16A11 antibody (1/500 dilution, Invitrogen) for post-mitotic neurons or rabbit monoclonal anti-active caspase-3 (1∶200, Abcam) to detect the initiation of apoptosis. Goat anti-mouse IgG HRP or goat anti-rabbit IgG HRP (Roche) was used to detect the primary antibodies and DAB was used as a substrate for secondary antibody-conjugated HRP (Amresco). Embryos were mounted with Vectashield mouting medium (Vector Laboratories, Inc.).

### Quantitative Analysis

For quantitative real time PCR (qPCR), embryos were homogenized in TRIzol reagent (Invitrogen) and total RNA was extracted using a standard method. cDNA was synthesized from total RNA with random hexamer priming using RevertAid First Strand cDNA Synthesis Kit (Fermentas). qPCR was performed on an ABI StepOne™ Real-Time PCR System (Applied Biosystems) with SYBR green fluorescent label (Fermentas). Primers for *neurogenein1* (F: 5′-CGCACACGGATGATGAAGACTCGCG-3′; R: 5′-CGGTTCTTCTTCACGACGTGCACAGTGG-3′), *deltaA* (F: 5′-ACCGGGTGAAGCTTGTGAAC-3′; R: 5′-CGTCATGCYCGTCCAGAAGTT-3′), *her8a* (F: 5′-GTAACGGGGAGACGCGTCTGCAGCG-3′; R: 5′-GATTATTCCCACGATGACGGCGGCG-3′) and *elavl3* (F: 5′-ACTGAGGAGTGGTATCGCTCAAA-3′; R: 5′-AGACCCACGGAGAGATTCCA-3′) were used. Gene expression levels were normalized to *gapdh* and assessed using the comparative CT (40 cycles) according to the manufacturer’s instructions (Applied Biosystems).

For Western blot analysis, embryos were homogenized in SDS lysis buffer. 60 µg were loaded on 12% SDS polyacrylamide gel and transferred to a PVDF membrane and detected with anti-HuC/HuD monoclonal antibody (1∶1000, Invitrogen). After washes, membranes were incubated with goat anti-Mouse HRP-conjugated secondary Ab (Chemicon) and developed with ECL (Millipore). Band intensities were quantified using Multi Gaugre analysis software.

Statistical analysis was performed by student’s t-test using Microsoft Excel® 2007. The significance level was set at P<0.05. All Reaction was performed in triplicate for each sample.

## Supporting Information

Figure S1 **Alignment of Akt1 homologs and synteny comparison.** (A) Amino acid alignment of human, mouse, chicken, and zebrafish AKT1/Akt1 sequences. Residues that were identical in all proteins are marked with black boxes while similarity is shown by gray boxes. The pleckstrin homology (PH) domain, kinase domain, and hydrophobic motif are indicated. The intron positions are marked with arrowheads and the conserved phosphorylation sites with asterisks. (B) Phylogenetic tree of the Akt protein family. Full coding protein sequences were used for each family member. Trees were calculated using bootstrapping with 100 replicates. The phylogram shows only the sequence relationships; it does not imply absolute sequence ancestry because no ancestral relationship was assumed in the initial alignments. Genes are not drawn to scale. The initial letter “h” denotes human, “m” is mouse, “c” is chicken, and “z” is zebrafish.
(TIF)

Figure S2 **Injection of** ***akt1*** **morpholinos effectively knocks down Akt1 protein production.** (A) Schematic representation showing the genomic organization of the *akt1* gene. The regions targeted by translational-blocking (MO1) and splice-blocking (MO2) morpholinos are shown. (B) The efficacy of MO2 was validated by RT-PCR using the primers indicated in A. Wild-type *akt1* mRNA produced a 1425 bp PCR product, whereas alternatively spliced transcripts from morphant embryos yielded a 1206 bp fragment. (C) The mis-splicing event resulted in the loss of exon 10, which was confirmed by sequencing of the PCR product in B. The mis-splicing event resulted in a premature stop codon in exon 11 (red box). (D) An mRNA encoding a morpholino control construct (*5′ akt1-EGFP*) was injected with the control or the *akt1* morpholino. Embryos coinjected with *5′ akt1-EGFP* mRNA and the control morpholino displayed strong EGFP expression (left panel). By contrast, the EGFP signal was abolished in embryos coinjected with *5′ akt1-EGFP* mRNA and the *akt1* morpholino (right panel).
(TIF)

Figure S3 **Embryos injected with a five-base mismatch morpholino show unaltered expression of neural markers.** The specificity of the *akt1* morpholinos was confirmed by injection with a morpholino with a five-base mismatch relative to MO2. Injection of this morpholino resulted in unaltered expression of neural markers showing by *in situ* hybridization (A) and quantitatively confirmed by qPCR (B).
(TIF)

## References

[1] Staal SP, Hartley JW, Rowe WP (1977) Isolation of transforming murine leukemia viruses from mice with a high incidence of spontaneous lymphoma. Proc Natl Acad Sci U S A 74: 3065–3067. 197531 10.1073/pnas.74.7.3065 PMC431413

[2] Coffer PJ, Woodgett JR (1991) Molecular cloning and characterisation of a novel putative protein-serine kinase related to the cAMP-dependent and protein kinase C families. Eur J Biochem 201: 475–481. 1718748 10.1111/j.1432-1033.1991.tb16305.x

[3] Jones PF, Jakubowicz T, Pitossi FJ, Maurer F, Hemmings BA (1991) Molecular cloning and identification of a serine/threonine protein kinase of the second-messenger subfamily. Proc Natl Acad Sci U S A 88: 4171–4175. 1851997 10.1073/pnas.88.10.4171 PMC51620

[4] Manning BD, Cantley LC (2007) AKT/PKB signaling: navigating downstream. Cell 129: 1261–1274. 17604717 10.1016/j.cell.2007.06.009 PMC2756685

[5] Franke TF (2008) PI3K/Akt: getting it right matters. Oncogene 27: 6473–6488. 18955974 10.1038/onc.2008.313

[6] Kumar CC, Madison V (2005) AKT crystal structure and AKT-specific inhibitors. Oncogene 24: 7493–7501. 16288296 10.1038/sj.onc.1209087

[7] Stambolic V, Woodgett JR (2006) Functional distinctions of protein kinase B/Akt isoforms defined by their influence on cell migration. Trends Cell Biol 16: 461–466. 16870447 10.1016/j.tcb.2006.07.001

[8] Saji M, Narahara K, McCarty SK, Vasko VV, La Perle KM, et al (2011) Akt1 deficiency delays tumor progression, vascular invasion, and distant metastasis in a murine model of thyroid cancer. Oncogene 30: 4307–4315. 21532616 10.1038/onc.2011.136 PMC3151477

[9] Martelli AM, Nyakern M, Tabellini G, Bortul R, Tazzari PL, et al (2006) Phosphoinositide 3-kinase/Akt signaling pathway and its therapeutical implications for human acute myeloid leukemia. Leukemia 20: 911–928. 16642045 10.1038/sj.leu.2404245

[10] Holland EC, Celestino J, Dai C, Schaefer L, Sawaya RE, et al (2000) Combined activation of Ras and Akt in neural progenitors induces glioblastoma formation in mice. Nat Genet 25: 55–57. 10802656 10.1038/75596

[11] Carpten JD, Faber AL, Horn C, Donoho GP, Briggs SL, et al (2007) A transforming mutation in the pleckstrin homology domain of AKT1 in cancer. Nature 448: 439–444. 17611497 10.1038/nature05933

[12] Lindhurst MJ, Sapp JC, Teer JK, Johnston JJ, Finn EM, et al (2011) A mosaic activating mutation in AKT1 associated with the Proteus syndrome. N Engl J Med 365: 611–619. 21793738 10.1056/NEJMoa1104017 PMC3170413

[13] Emamian ES, Hall D, Birnbaum MJ, Karayiorgou M, Gogos JA (2004) Convergent evidence for impaired AKT1-GSK3beta signaling in schizophrenia. Nat Genet 36: 131–137. 14745448 10.1038/ng1296

[14] Dudek H, Datta SR, Franke TF, Birnbaum MJ, Yao R, et al (1997) Regulation of neuronal survival by the serine-threonine protein kinase Akt. Science 275: 661–665. 9005851 10.1126/science.275.5300.661

[15] Namikawa K, Honma M, Abe K, Takeda M, Mansur K, et al (2000) Akt/protein kinase B prevents injury-induced motoneuron death and accelerates axonal regeneration. J Neurosci 20: 2875–2886. 10751440 10.1523/JNEUROSCI.20-08-02875.2000 PMC6772200

[16] Ries V, Henchcliffe C, Kareva T, Rzhetskaya M, Bland R, et al (2006) Oncoprotein Akt/PKB induces trophic effects in murine models of Parkinson’s disease. Proc Natl Acad Sci U S A 103: 18757–18762. 17116866 10.1073/pnas.0606401103 PMC1654135

[17] Yuan J, Yankner BA (2000) Apoptosis in the nervous system. Nature 407: 802–809. 11048732 10.1038/35037739

[18] Brunet A, Datta SR, Greenberg ME (2001) Transcription-dependent and -independent control of neuronal survival by the PI3K-Akt signaling pathway. Curr Opin Neurobiol 11: 297–305. 11399427 10.1016/s0959-4388(00)00211-7

[19] Sinor AD, Lillien L (2004) Akt-1 expression level regulates CNS precursors. J Neurosci 24: 8531–8541. 15456827 10.1523/JNEUROSCI.1470-04.2004 PMC6729906

[20] Enomoto A, Murakami H, Asai N, Morone N, Watanabe T, et al (2005) Akt/PKB regulates actin organization and cell motility via Girdin/APE. Dev Cell 9: 389–402. 16139227 10.1016/j.devcel.2005.08.001

[21] Zhou FQ, Snider WD (2006) Intracellular control of developmental and regenerative axon growth. Philos Trans R Soc Lond B Biol Sci 361: 1575–1592. 16939976 10.1098/rstb.2006.1882 PMC1664665

[22] Vojtek AB, Taylor J, DeRuiter SL, Yu JY, Figueroa C, et al (2003) Akt regulates basic helix-loop-helix transcription factor-coactivator complex formation and activity during neuronal differentiation. Mol Cell Biol 23: 4417–4427. 12808085 10.1128/MCB.23.13.4417-4427.2003 PMC164860

[23] Oishi K, Watatani K, Itoh Y, Okano H, Guillemot F, et al (2009) Selective induction of neocortical GABAergic neurons by the PDK1-Akt pathway through activation of Mash1. Proc Natl Acad Sci U S A 106: 13064–13069. 19549840 10.1073/pnas.0808400106 PMC2722283

[24] Bang OS, Park EK, Yang SI, Lee SR, Franke TF, et al (2001) Overexpression of Akt inhibits NGF-induced growth arrest and neuronal differentiation of PC12 cells. J Cell Sci 114: 81–88. 11112692 10.1242/jcs.114.1.81

[25] Dottori M, Leung J, Turnley AM, Pebay A (2008) Lysophosphatidic acid inhibits neuronal differentiation of neural stem/progenitor cells derived from human embryonic stem cells. Stem Cells 26: 1146–1154. 18308941 10.1634/stemcells.2007-1118

[26] Yang ZZ, Tschopp O, Di-Poi N, Bruder E, Baudry A, et al (2005) Dosage-dependent effects of Akt1/protein kinase Balpha (PKBalpha) and Akt3/PKBgamma on thymus, skin, and cardiovascular and nervous system development in mice. Mol Cell Biol 25: 10407–10418. 16287854 10.1128/MCB.25.23.10407-10418.2005 PMC1291243

[27] Chen WS, Xu PZ, Gottlob K, Chen ML, Sokol K, et al (2001) Growth retardation and increased apoptosis in mice with homozygous disruption of the Akt1 gene. Genes Dev 15: 2203–2208. 11544177 10.1101/gad.913901 PMC312770

[28] Cho H, Thorvaldsen JL, Chu Q, Feng F, Birnbaum MJ (2001) Akt1/PKBalpha is required for normal growth but dispensable for maintenance of glucose homeostasis in mice. J Biol Chem 276: 38349–38352. 11533044 10.1074/jbc.C100462200

[29] Lai WS, Xu B, Westphal KG, Paterlini M, Olivier B, et al (2006) Akt1 deficiency affects neuronal morphology and predisposes to abnormalities in prefrontal cortex functioning. Proc Natl Acad Sci U S A 103: 16906–16911. 17077150 10.1073/pnas.0604994103 PMC1636552

[30] Peng Y, Jiang BH, Yang PH, Cao Z, Shi X, et al (2004) Phosphatidylinositol 3-kinase signaling is involved in neurogenesis during Xenopus embryonic development. J Biol Chem 279: 28509–28514. 15123704 10.1074/jbc.M402294200

[31] Alessi DR, Andjelkovic M, Caudwell B, Cron P, Morrice N, et al (1996) Mechanism of activation of protein kinase B by insulin and IGF-1. EMBO J 15: 6541–6551. 8978681 PMC452479

[32] Shao Z, Bhattacharya K, Hsich E, Park L, Walters B, et al (2006) c-Jun N-terminal kinases mediate reactivation of Akt and cardiomyocyte survival after hypoxic injury in vitro and in vivo. Circ Res 98: 111–118. 16306447 10.1161/01.RES.0000197781.20524.b9

[33] Taylor JS, Van de Peer Y, Braasch I, Meyer A (2001) Comparative genomics provides evidence for an ancient genome duplication event in fish. Philos Trans R Soc Lond B Biol Sci 356: 1661–1679. 11604130 10.1098/rstb.2001.0975 PMC1088543

[34] Burke RE (2007) Inhibition of mitogen-activated protein kinase and stimulation of Akt kinase signaling pathways: Two approaches with therapeutic potential in the treatment of neurodegenerative disease. Pharmacol Ther 114: 261–277. 17399794 10.1016/j.pharmthera.2007.02.002 PMC1964795

[35] Robu ME, Larson JD, Nasevicius A, Beiraghi S, Brenner C, et al (2007) p53 activation by knockdown technologies. PLoS Genet 3: e78. 17530925 10.1371/journal.pgen.0030078 PMC1877875

[36] Takamiya M, Campos-Ortega JA (2006) Hedgehog signalling controls zebrafish neural keel morphogenesis via its level-dependent effects on neurogenesis. Dev Dyn 235: 978–997. 16502420 10.1002/dvdy.20720

[37] Androutsellis-Theotokis A, Leker RR, Soldner F, Hoeppner DJ, Ravin R, et al (2006) Notch signalling regulates stem cell numbers in vitro and in vivo. Nature 442: 823–826. 16799564 10.1038/nature04940

[38] Louvi A, Artavanis-Tsakonas S (2006) Notch signalling in vertebrate neural development. Nat Rev Neurosci 7: 93–102. 16429119 10.1038/nrn1847

[39] Chitnis AB (1995) The role of Notch in lateral inhibition and cell fate specification. Mol Cell Neurosci 6: 311–321. 8742272

[40] Cheng YC, Amoyel M, Qiu X, Jiang YJ, Xu Q, et al (2004) Notch activation regulates the segregation and differentiation of rhombomere boundary cells in the zebrafish hindbrain. Dev Cell 6: 539–550. 15068793 10.1016/s1534-5807(04)00097-8

[41] Chung PC, Lin WS, Scotting PJ, Hsieh FY, Wu HL, et al (2011) Zebrafish Her8a is activated by Su(H)-dependent Notch signaling and is essential for the inhibition of neurogenesis. PLoS One 6: e19394. 21541299 10.1371/journal.pone.0019394 PMC3082574

[42] Zhang J, Fukuhara S, Sako K, Takenouchi T, Kitani H, et al (2011) Angiopoietin-1/Tie2 signal augments basal Notch signal controlling vascular quiescence by inducing delta-like 4 expression through AKT-mediated activation of beta-catenin. J Biol Chem 286: 8055–8066. 21212269 10.1074/jbc.M110.192641 PMC3048692

[43] Ma J, Meng Y, Kwiatkowski DJ, Chen X, Peng H, et al (2010) Mammalian target of rapamycin regulates murine and human cell differentiation through STAT3/p63/Jagged/Notch cascade. J Clin Invest 120: 103–114. 20038814 10.1172/JCI37964 PMC2798675

[44] Calzavara E, Chiaramonte R, Cesana D, Basile A, Sherbet GV, et al (2008) Reciprocal regulation of Notch and PI3K/Akt signalling in T-ALL cells in vitro. J Cell Biochem 103: 1405–1412. 17849443 10.1002/jcb.21527

[45] Zhao N, Guo Y, Zhang M, Lin L, Zheng Z (2010) Akt-mTOR signaling is involved in Notch-1-mediated glioma cell survival and proliferation. Oncol Rep 23: 1443–1447. 20372862 10.3892/or_00000782

[46] Palomero T, Sulis ML, Cortina M, Real PJ, Barnes K, et al (2007) Mutational loss of PTEN induces resistance to NOTCH1 inhibition in T-cell leukemia. Nat Med 13: 1203–1210. 17873882 10.1038/nm1636 PMC2600418

[47] Palomero T, Dominguez M, Ferrando AA (2008) The role of the PTEN/AKT Pathway in NOTCH1-induced leukemia. Cell Cycle 7: 965–970. 18414037 10.4161/cc.7.8.5753 PMC2600414

[48] Jiang YJ, Brand M, Heisenberg CP, Beuchle D, Furutani-Seiki M, et al (1996) Mutations affecting neurogenesis and brain morphology in the zebrafish, Danio rerio. Development 123: 205–216. 9007241 10.1242/dev.123.1.205

[49] Itoh M, Kim CH, Palardy G, Oda T, Jiang YJ, et al (2003) Mind bomb is a ubiquitin ligase that is essential for efficient activation of Notch signaling by Delta. Dev Cell 4: 67–82. 12530964 10.1016/s1534-5807(02)00409-4

[50] Wettstein DA, Turner DL, Kintner C (1997) The Xenopus homolog of Drosophila Suppressor of Hairless mediates Notch signaling during primary neurogenesis. Development 124: 693–702. 9043084 10.1242/dev.124.3.693

[51] Beaulieu JM, Gainetdinov RR, Caron MG (2007) The Akt-GSK-3 signaling cascade in the actions of dopamine. Trends Pharmacol Sci 28: 166–172. 17349698 10.1016/j.tips.2007.02.006

[52] Wang L, Zhang ZG, Zhang RL, Jiao ZX, Wang Y, et al (2006) Neurogenin 1 mediates erythropoietin enhanced differentiation of adult neural progenitor cells. J Cereb Blood Flow Metab 26: 556–564. 16136056 10.1038/sj.jcbfm.9600215

[53] Bedogni B, Warneke JA, Nickoloff BJ, Giaccia AJ, Powell MB (2008) Notch1 is an effector of Akt and hypoxia in melanoma development. J Clin Invest 118: 3660–3670. 18924608 10.1172/JCI36157 PMC2567838

[54] McKenzie G, Ward G, Stallwood Y, Briend E, Papadia S, et al (2006) Cellular Notch responsiveness is defined by phosphoinositide 3-kinase-dependent signals. BMC Cell Biol 7: 10. 16507111 10.1186/1471-2121-7-10 PMC1403772

[55] Song J, Park S, Kim M, Shin I (2008) Down-regulation of Notch-dependent transcription by Akt in vitro. FEBS Lett 582: 1693–1699. 18440314 10.1016/j.febslet.2008.04.024

[56] Jo HS, Kang KH, Joe CO, Kim JW (2012) Pten coordinates retinal neurogenesis by regulating Notch signalling. EMBO J 31: 817–828. 22258620 10.1038/emboj.2011.443 PMC3280546

[57] Liu ZJ, Xiao M, Balint K, Smalley KS, Brafford P, et al (2006) Notch1 signaling promotes primary melanoma progression by activating mitogen-activated protein kinase/phosphatidylinositol 3-kinase-Akt pathways and up-regulating N-cadherin expression. Cancer Res 66: 4182–4190. 16618740 10.1158/0008-5472.CAN-05-3589

[58] Cheng Y, Cheung M, Abu-Elmagd MM, Orme A, Scotting PJ (2000) Chick sox10, a transcription factor expressed in both early neural crest cells and central nervous system. Brain Res Dev Brain Res 121: 233–241. 10876038 10.1016/s0165-3806(00)00049-3

[59] Xu Q, Alldus G, Holder N, Wilkinson DG (1995) Expression of truncated Sek-1 receptor tyrosine kinase disrupts the segmental restriction of gene expression in the Xenopus and zebrafish hindbrain. Development 121: 4005–4016. 8575301 10.1242/dev.121.12.4005

