# Supplementary figures and images for: Akt1 Mediates Neuronal Differentiation in Zebrafish via a Reciprocal Interaction with Notch Signaling
Source: PLoS One. 2013 Jan 14;8(1):e54262. doi: 10.1371/journal.pone.0054262 (PMC3544797; doi:10.1371/journal.pone.0054262)

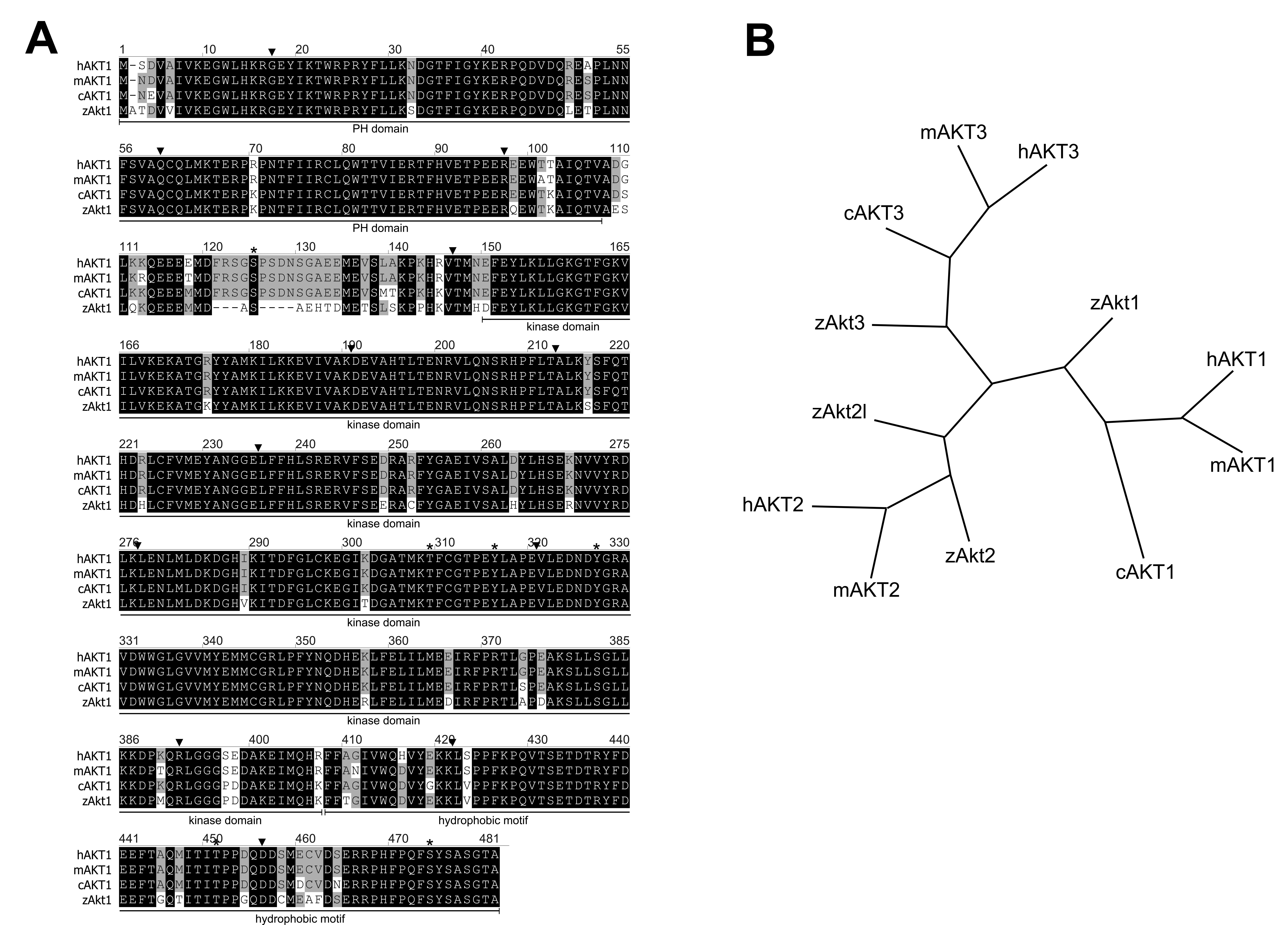

Supplement: Figure S1 — Alignment of Akt1 homologs and synteny comparison. (A) Amino acid alignment of human, mouse, chicken, and zebrafish AKT1/Akt1 sequences. Residues that were identical in all proteins are marked with black boxes while similarity is shown by gray boxes. The pleckstrin homology (PH) domain, kinase domain, and hydrophobic motif are indicated. The intron positions are marked with arrowheads and the conserved phosphorylation sites with asterisks. (B) Phylogenetic tree of the Akt protein family. Full coding protein sequences were used for each family member. Trees were calculated using bootstrapping with 100 replicates. The phylogram shows only the sequence relationships; it does not imply absolute sequence ancestry because no ancestral relationship was assumed in the initial alignments. Genes are not drawn to scale. The initial letter “h” denotes human, “m” is mouse, “c” is chicken, and “z” is zebrafish. (TIF) [file pone.0054262.s001.tif]

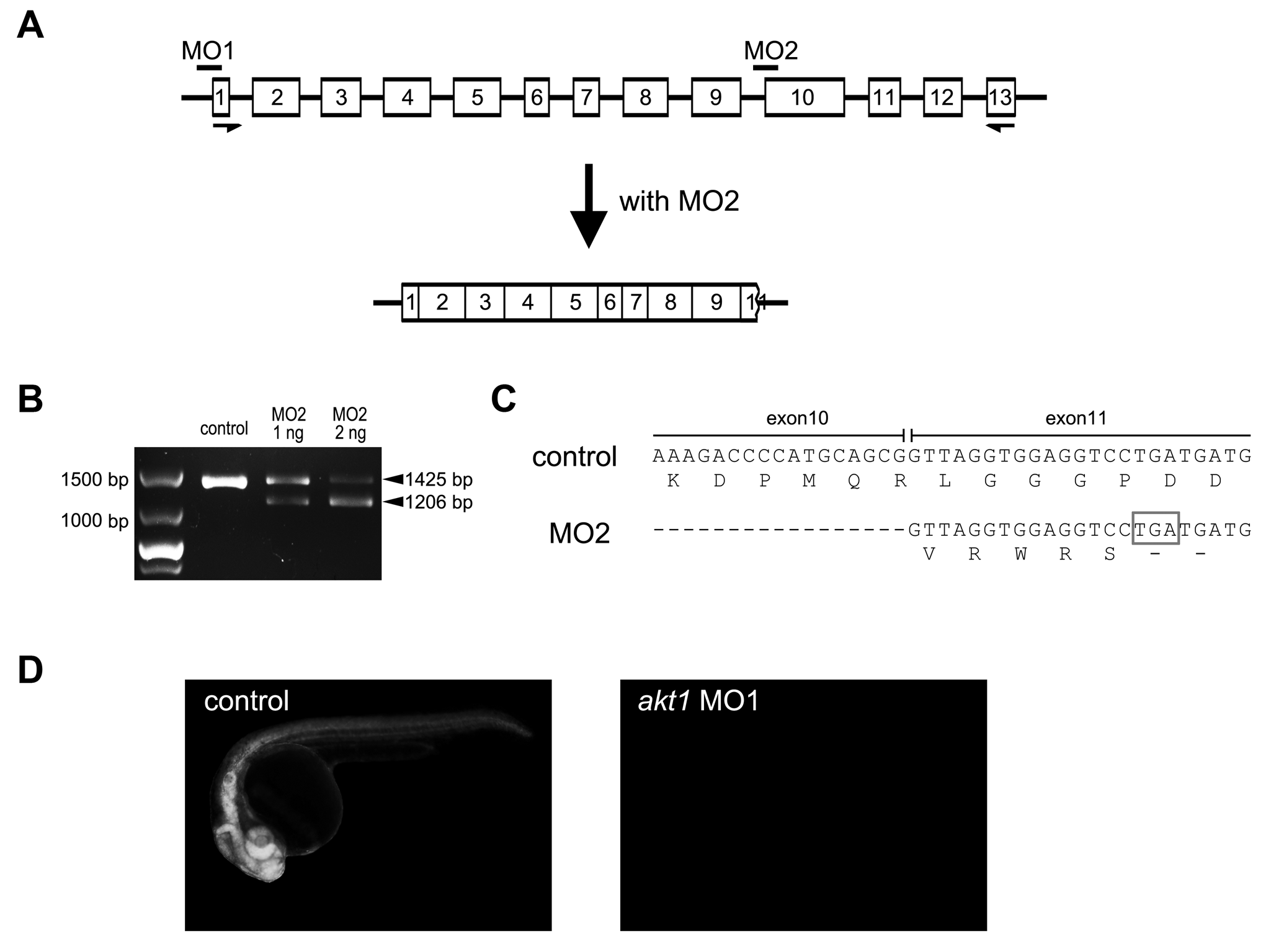

Supplement: Figure S2 — Injection of akt1 morpholinos effectively knocks down Akt1 protein production. (A) Schematic representation showing the genomic organization of the akt1 gene. The regions targeted by translational-blocking (MO1) and splice-blocking (MO2) morpholinos are shown. (B) The efficacy of MO2 was validated by RT-PCR using the primers indicated in A. Wild-type akt1 mRNA produced a 1425 bp PCR product, whereas alternatively spliced transcripts from morphant embryos yielded a 1206 bp fragment. (C) The mis-splicing event resulted in the loss of exon 10, which was confirmed by sequencing of the PCR product in B. The mis-splicing event resulted in a premature stop codon in exon 11 (red box). (D) An mRNA encoding a morpholino control construct (5′ akt1-EGFP) was injected with the control or the akt1 morpholino. Embryos coinjected with 5′ akt1-EGFP mRNA and the control morpholino displayed strong EGFP expression (left panel). By contrast, the EGFP signal was abolished in embryos coinjected with 5′ akt1-EGFP mRNA and the akt1 morpholino (right panel). (TIF) [file pone.0054262.s002.tif]

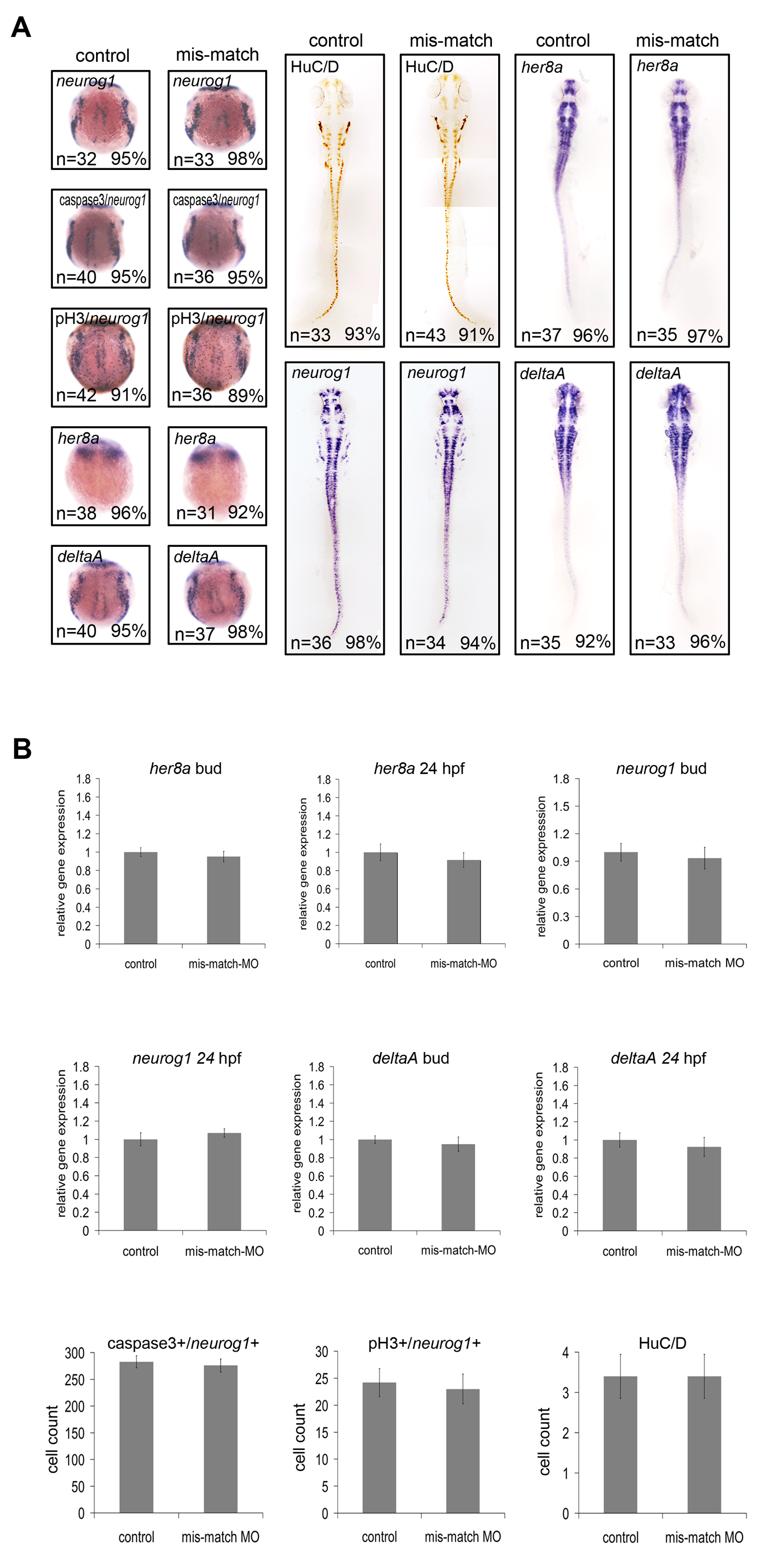

Supplement: Figure S3 — Embryos injected with a five-base mismatch morpholino show unaltered expression of neural markers. The specificity of the akt1 morpholinos was confirmed by injection with a morpholino with a five-base mismatch relative to MO2. Injection of this morpholino resulted in unaltered expression of neural markers showing by in situ hybridization (A) and quantitatively confirmed by qPCR (B). (TIF) [file pone.0054262.s003.tif]
